# Institutional barriers to assisted reproductive technology access in rural South Africa: a qualitative study of medical officers’ perspectives

**DOI:** 10.3389/fpubh.2026.1842990

**Published:** 2026-07-29

**Authors:** Nomfundo N. Mthembu, Betty C. Mubangizi

**Affiliations:** 1 School of Law, College of Law and Management Studies, University of KwaZulu-Natal, Durban, South Africa; 2 College of Law and Management Studies, University of KwaZulu-Natal, Durban, South Africa

**Keywords:** assisted reproductive technologies, barriers, health policy, reproductive health, rural health

## Abstract

**Background:**

Access to assisted reproductive technologies (ARTs) remains highly unequal globally, with rural populations facing significant geographic, financial, and institutional barriers to fertility care. In South Africa, despite constitutional guarantees of access to reproductive healthcare, advanced fertility services remain concentrated in urban centres and the private healthcare sector, limiting access for many rural communities.

**Methods:**

This qualitative study explored institutional barriers to ART access from the perspectives of medical officers working in the uMhlabuyalingana Local Municipality, KwaZulu-Natal, South Africa. Semi-structured interviews were conducted with 12 medical officers and analysed using thematic analysis. Institutional Theory provided the conceptual lens through which findings were interpreted.

**Results:**

Three major themes emerged from the analysis: (1) limited scope of infertility management in rural healthcare facilities, constrained by infrastructure, personnel, and resource limitations; (2) inadequate patient and provider education, resulting in low fertility awareness and delayed care-seeking; and (3) weak health system capacity, characterised by fragmented referral pathways, workforce shortages, and limited access to specialised fertility services. Participants described how these barriers collectively restrict the availability, accessibility, and continuity of infertility care for rural populations.

**Conclusion:**

The findings demonstrate that inequitable access to ART in rural South Africa is shaped not only by geographic distance but also by broader institutional arrangements that systematically disadvantage rural communities. Strengthening health system capacity, decentralising selected fertility services, improving referral systems, and integrating infertility education into primary healthcare are critical steps toward advancing reproductive justice and achieving equitable access to reproductive healthcare.

## 1 Introduction

Access to assisted reproductive technologies (ARTs) represents a critical dimension of reproductive healthcare, yet significant disparities persist globally, particularly affecting populations in rural and underserved regions (1, 2). In South Africa, Section 27 of the Constitution guarantees all persons access to healthcare, including reproductive health services. However, the reality for rural populations reveals substantial gaps between constitutional rights and lived experiences. Fertility services, including *in vitro* fertilisation (IVF), intrauterine insemination (IUI), and fertility preservation, remain concentrated in urban centres and are predominantly accessible through private healthcare systems (3). This geographic and economic concentration creates profound inequities, leaving rural residents, who constitute approximately 38% of South Africa’s population (4), effectively excluded from advanced reproductive healthcare.

The challenges facing rural populations seeking ARTs are multifaceted, encompassing geographic barriers, economic constraints, limited healthcare infrastructure, and cultural factors (5, 22). Rural district hospitals typically lack specialised fertility services, trained personnel, and diagnostic equipment necessary for comprehensive infertility management (6). Patients must travel long distances to urban centres, incurring substantial costs for transportation, accommodation, and treatment itself (7). These barriers are compounded by limited awareness of infertility causes, treatment options, and available services among both patients and medical officers in rural settings (8).

Despite growing recognition of reproductive health disparities, empirical research examining ART access from the perspective of rural healthcare providers remains limited, particularly in the South African context. Medical officers occupy a critical position at the intersection of health systems and patient experiences, offering unique insights into institutional barriers, resource constraints, and potential solutions. Understanding their perspectives is essential for developing evidence-based interventions that address systemic challenges rather than focusing solely on individual-level factors.

This study addresses this gap by examining the experiences and perspectives of medical officers working in rural district hospitals and private practices in the UMhlabuyalingana district of KwaZulu-Natal, South Africa. The primary research question guiding this investigation is: In light of advances in medical technologies such as assisted reproductive technologies, can patients in rural areas access these technologies, or are they left behind? Specifically, this study explores: (1) the current state of infertility management and treatment in rural healthcare facilities; (2) challenges faced by medical officers in treating and managing infertility; (3) challenges faced by individuals facing infertility in rural regions; and (4) how education and awareness of infertility have been addressed in rural healthcare settings.

Grounded in Institutional Theory (9), this research examines how regulative, normative, and cultural-cognitive institutional pillars shape access to ART in rural South Africa. Institutional Theory provides a robust framework for understanding how formal rules, professional norms, and taken-for-granted assumptions create and perpetuate healthcare inequities. By analysing institutional barriers through this lens, the study contributes to both theoretical understanding and practical policy development aimed at achieving reproductive justice in underserved communities.

## 2 Research methods

### 2.1 Study design and setting

This study adopted an exploratory qualitative research design to examine institutional barriers to assisted reproductive technology (ART) access from the perspectives of medical officers working in a rural South African setting. An exploratory qualitative approach was considered appropriate because limited empirical research has examined how frontline healthcare providers experience and navigate barriers to infertility management and ART access in rural contexts. Semi-structured interviews were used to generate in-depth insights into participants’ experiences and perceptions. South Africa’s reproductive health system provides services through both public and private healthcare sectors. However, infertility care and assisted reproductive technologies (ARTs) remain concentrated within specialised urban centres and predominantly private healthcare facilities, creating geographic and financial barriers for many rural populations (2, 21). These challenges are reinforced by South Africa’s dual healthcare system, in which a resource-constrained public sector serves the majority of the population while advanced fertility services are largely located within the private sector.

The study was conducted in uMhlabuyalingana Local Municipality, located within uMkhanyakude District in northern KwaZulu-Natal. The municipality was purposively selected because it is among the most remote and socio-economically disadvantaged rural areas in South Africa, characterised by widespread poverty, dispersed settlement patterns, limited transport infrastructure, and constrained access to specialised healthcare services (10). The district is served by public hospitals and private general practices but has no specialised fertility clinics, requiring patients to rely on referral pathways to urban centres for advanced fertility services. These characteristics make the municipality an appropriate setting for examining institutional barriers to ART access in rural communities.

### 2.2 Participants and recruitment

Participants were recruited using a total population sampling approach. All medical officers practising in public hospitals and private healthcare facilities within the uMhlabuyalingana district were eligible to participate, irrespective of their years of experience or clinical specialisation. Recruitment was undertaken through direct contact with healthcare facilities, including communication with facility managers and the distribution of study information sheets to eligible medical officers. Medical officers who expressed willingness to participate were contacted directly by the researcher to arrange interviews.

Twelve medical officers agreed to participate in the study, representing both public-sector physicians working in district hospitals and private practitioners within the district. Given the relatively small number of medical officers practising in this rural setting, all available and consenting participants were included in the study. Data collection continued until no substantially new themes emerged from successive interviews, indicating thematic saturation.

### 2.3 Data collection

Data were collected through semi-structured interviews conducted between 2024 and 2025. Interviews were conducted either in person, remotely using Zoom, Microsoft Teams, or telephone calls, depending on participant preference and logistical considerations. The interview guide was developed based on the study’s research questions and covered five main areas: (1) current practices in infertility management and treatment; (2) challenges faced by medical officers in providing fertility care; (3) challenges faced by patients seeking fertility services; (4) education and awareness initiatives related to infertility; and (5) recommendations for improving ART access from a rural medicine perspective.

Interviews lasted approximately 45–60 min and were audio-recorded with participant consent. The semi-structured format allowed for flexibility to explore emerging themes while ensuring consistent coverage of key topics across all interviews. Participants were assured of confidentiality, and no personal patient data were collected.

### 2.4 Theoretical framework: institutional theory and healthcare access

Institutional Theory offers a powerful lens for understanding how organisational fields, such as healthcare systems, are shaped by formal and informal rules, norms, and cognitive frameworks that guide behaviour and structure access to resources (9). This theoretical perspective is particularly relevant for examining ART access disparities, as it directs attention beyond individual-level factors to the broader institutional arrangements that enable or constrain healthcare delivery in different geographic and social contexts.

Scott's (9) framework identifies three institutional pillars that collectively shape organisational behaviour and outcomes: regulative, normative, and cultural-cognitive. The *regulative pillar* encompasses formal rules, laws, policies, and regulations that govern healthcare provision. In the context of ART access, regulative elements include national health policies, insurance coverage mandates, licensing requirements for fertility clinics, and resource allocation decisions (11). In South Africa, the absence of comprehensive public funding for ART and the concentration of services in private, urban facilities reflect regulative choices that systematically disadvantage rural populations.

The *normative pillar* refers to professional standards, values, and expectations that define appropriate behaviour within healthcare systems. This includes clinical guidelines, professional training curricula, and shared understandings about which health conditions merit priority and resource investment. The relative neglect of infertility in rural health programming, compared to other reproductive health issues, reflects normative priorities that may not recognise fertility care as essential healthcare, as discussed by Peterson (12).

The *cultural-cognitive pillar* encompasses taken-for-granted assumptions, shared beliefs, and cognitive frameworks that shape how actors interpret and respond to their environment. In rural healthcare contexts, this includes assumptions about which services are feasible or appropriate for rural settings, beliefs about patient demand for fertility services, and cognitive schemas that associate advanced reproductive technologies exclusively with urban, affluent populations (5).

These three pillars interact to create institutional environments that profoundly shape healthcare access. Geographic disparities in ART access reflect not merely the physical distribution of clinics, but deeper institutional logics that concentrate specialised services in metropolitan areas (1, 13). Studies from China, the United States, and Australia demonstrate that even when ART services exist, residential proximity to clinics independently predicts utilisation, suggesting that institutional arrangements privilege those already advantaged by geography (1, 14, 15, 22).

Institutional Theory also highlights the concept of *institutional isomorphism*—the tendency for organisations within a field to become increasingly similar over time through coercive, mimetic, and normative pressures (16). In healthcare, this can result in the replication of urban-centric service models that are poorly suited to rural contexts. For example, the concentration of ART services in specialised, high-volume clinics reflects an institutional model optimised for urban settings but creates access barriers for geographically dispersed populations (7).

Furthermore, Institutional Theory draws attention to institutional work - the purposive actions of individuals and organisations to create, maintain, or disrupt institutions (23). Medical officers in rural settings engage in institutional work when they advocate for policy changes, develop innovative service delivery models, or challenge normative assumptions about rural healthcare capabilities. Understanding this institutional work is crucial for identifying pathways toward more equitable ART access.

The theory informed the interpretation of themes generated through thematic analysis by enabling exploration of how formal structures, professional norms, and prevailing assumptions shaped access to ART services in rural healthcare settings.

### 2.5 Data analysis

Interview recordings were transcribed verbatim by a trained research assistant. Transcripts were analysed using thematic analysis, following the six-phase approach outlined by Braun and Clarke (24). The analysis process involved: (1) familiarization with the data through repeated reading of transcripts; (2) generation of initial codes identifying features of the data relevant to the research questions; (3) searching for themes by collating codes into potential patterns; (4) reviewing themes to ensure they accurately reflected the coded data and overall dataset; (5) defining and naming themes to capture their essence; and (6) producing the final analysis with illustrative quotations.

The analysis followed an inductive thematic approach. Themes were generated from the data and subsequently interpreted through the lens of Institutional Theory, as described in Section 3.4. Three major themes emerged from the analysis, corresponding to the study’s focus on institutional barriers to ART access: (1) limited scope of infertility management in rural facilities; (2) inadequate patient and provider education; and (3) weak health system capacity. These themes are presented in the Results section, supported by evidence from participant interviews.

### 2.6 Ethical considerations

The study received ethical approval from the University of KwaZulu-Natal’s Biomedical Research Ethics Committee. All participants provided informed consent prior to participation. Data were stored securely in password-protected digital formats and will be retained for 5 years in accordance with institutional policy, after which they will be permanently deleted. Participant confidentiality was maintained throughout the research process, with all identifying information removed from transcripts and replaced with participant numbers in reporting.

### 2.7 Trustworthiness

Several strategies were employed to enhance the trustworthiness of findings. Credibility was established through prolonged engagement with the data, peer debriefing, and member checking where feasible. Dependability was ensured through detailed documentation of the research process and decision-making. Transferability was supported by providing a thick description of the research context, participants, and findings. Confirmability was enhanced through reflexive practice and maintaining an audit trail of analytical decisions.

## 3 Results

The analysis of interview data revealed three major themes reflecting institutional barriers to ART access in rural South Africa. These themes illuminate how regulative gaps, normative priorities, and cultural-cognitive assumptions embedded in healthcare systems create systematic disadvantages for rural populations seeking fertility care. Each theme is presented below with supporting evidence from participant interviews and connections to the broader literature on ART access disparities.

### 3.1 Theme 1: limited scope of infertility Management in Rural Facilities

The first central theme captures the profound constraints on infertility services available in rural district hospitals and practices. Participants consistently described their facilities as capable of providing only preliminary diagnostic investigations, with no capacity for therapeutic interventions or advanced fertility treatments. This limitation reflects institutional arrangements that concentrate specialised reproductive healthcare in urban tertiary centres while relegating rural facilities to basic screening and referral functions.

#### 3.1.1 Basic investigations only

Medical officers emphasised that infertility management in their settings was restricted to foundational assessments such as hormonal blood tests, ultrasounds, and hysterosalpingograms (HSGs). These procedures serve diagnostic purposes, identifying potential reproductive issues, but do not constitute treatment. As Participant 2 explained:

“So, currently, very little we can, obviously, do the 21-day hormones to see if they are ovulating, and we can do a hysterosalpingogram, so that is basically injecting contrast through the cervix to see if the tubes are open... That is really as far as it can go.”

This limitation was echoed across interviews. Participant 3 noted:

“Usually we do bloods just to check the hormones, uhmm, what else? We do ultrasounds and check for STIs, but for further investigations we refer to Queen Nandi, and they manage the rest.”

Similarly, Participant 7 stated:

“Currently, we do not really treat. Like we send the patient to an ultrasound to check any blocked tubes or fibroids or anything, then after the ultrasound, we do a hormonal level check.”

Male fertility investigations were particularly limited, as participant 12 explained:

“The male usually just does a sperm count only. There are challenges in doing the sperm counts because the closest centre will be in Empangeni or Richards Bay.”

This geographic barrier to even basic male fertility assessment reflects broader patterns of service concentration documented internationally (7).

These findings align with research from other rural contexts demonstrating that primary care facilities lack the infrastructure, equipment, and specialised personnel necessary for comprehensive fertility care (6, 8). In sub-Saharan Africa specifically, studies have documented the scarcity of ART clinics and the concentration of services in major urban centres, leaving rural populations dependent on referral systems that often fail to provide continuity of care (2).

#### 3.1.2 Absence of advanced treatments

Beyond basic diagnostics, participants unanimously reported the complete absence of therapeutic fertility interventions in their facilities. No hormonal treatments, intrauterine insemination, or assisted reproductive technologies were available locally.

Participant 1 stated:

“We do not have IVF here. We do not have the resources, we do not have the expertise.”

Participant 4 added:

“We cannot do anything beyond the basics. If they need actual treatment, they have to go to Durban or Johannesburg.”

This institutional arrangement creates a fundamental inequity: while urban residents can access comprehensive fertility care within their communities, rural patients must travel hundreds of kilometres to tertiary centres, incurring substantial costs and disruption to their lives. The absence of even intermediate-level treatments, such as ovulation induction or basic hormonal therapies, reflects normative assumptions about which services are appropriate for rural settings and regulative decisions about resource allocation that systematically disadvantage rural populations.

International evidence demonstrates that geographic concentration of ART services is a global phenomenon, with clinics clustered in metropolitan areas even in high-income countries with supportive health policies (13, 14, 22). In China, only 10.1% of approved ART institutions offer comprehensive services, with significant geographic disparities favouring eastern regions (22). In the United States, approximately 25% of the population lacks reasonable access to ART services, predominantly in rural areas (11). Australia, despite universal health insurance and public subsidies for ART, exhibits persistent geographic inequities, with residential proximity to clinics independently predicting treatment utilisation (14).

#### 3.1.3 Heavy reliance on referral systems

Given the absence of local treatment capacity, rural providers depend entirely on referral pathways to tertiary hospitals. However, participants described these referral systems as fragmented, unclear, and often ineffective.

Participant 6 explained:

“So, there is still a lot of stigma to get rid of A LOT. Except for physicians and patient education there must be improvement of referral pathways to facilities which have those or who offer those services.”

The referral process itself creates multiple barriers. Patients must navigate unfamiliar healthcare systems in distant cities, often without clear guidance about where to go, whom to see, or what to expect. Financial barriers compound these challenges, as patients must cover transportation, accommodation, and treatment costs. Many patients are lost to follow-up after referral, never accessing the specialised care they need.

This finding resonates with research documenting how fragmented healthcare pathways and poor care coordination undermine access to reproductive health services in rural settings (5, 8). In Australia, rural women seeking fertility care face disrupted continuity of care due to fly-in-fly-out practitioners and limited service availability at satellite clinics (5). In the United States, Veterans seeking IVF services report poor communication about benefits and a lack of comprehensive care coordination, particularly affecting those in rural areas (17).

### 3.2 Theme 2: inadequate patient and provider education

The second central theme highlights profound gaps in knowledge and awareness about infertility, its causes, and available treatments among both patients and medical officers in rural settings. These educational deficits reflect normative priorities within health systems that have historically under-emphasised fertility care, particularly in resource-constrained contexts. The absence of systematic education initiatives perpetuates misconceptions, delays care-seeking, and contributes to preventable infertility.

#### 3.2.1 Limited patient awareness

Participants consistently described low levels of awareness among rural patients regarding infertility causes, risk factors, and treatment options. Participant 10 emphasised:

“Education, education, education... tell them about STIs, ovulation cycle, alcohol, smoking... the general public doesn't know the basics.”

This knowledge gap extends to fundamental reproductive health concepts, including the relationship between sexually transmitted infections and infertility, the impact of lifestyle factors on fertility, and the existence of medical interventions for infertility. The consequences of limited awareness are significant. Patients often delay seeking care until they have been trying to conceive for many years, missing opportunities for earlier intervention when treatment may be more effective and less costly. Participant 4 commented:

“Yes, teach them... a person shouldn't come when they are a 40-yearold for the first time.”

This late presentation reflects not only individual knowledge gaps but also the absence of systematic public health education about reproductive health across the lifespan. Participant 8 highlighted specific medical causes of infertility that should be addressed through education:

“Fibroids, PID, ectopic pregnancies... infections are a big player... those are the things we should be teaching.”

The emphasis on preventable causes, particularly pelvic inflammatory disease resulting from untreated sexually transmitted infections, underscores the potential for education to reduce infertility incidence, not merely improve treatment access.

These findings align with research documenting low awareness of infertility and ART services in rural and underserved populations globally (6, 8). In sub-Saharan Africa, poor policy awareness and limited public education about fertility problems contribute to delayed care-seeking and reliance on traditional rather than biomedical approaches (2). In Brazil, inadequate information about reproductive health services and limited health literacy create barriers to accessing ART, particularly for low-income populations (18).

#### 3.2.2 Provider knowledge gaps

Beyond patient education, participants acknowledged gaps in their own knowledge and training regarding infertility management. Participant 6 stated:

“My first recommendation is to teach me how to do it. Second, give me resources and improve referral pathways.”

This candid acknowledgement reflects the reality that medical training in South Africa, as in many countries, provides limited exposure to reproductive endocrinology and fertility medicine, particularly for physicians who will practice in rural or primary care settings.

The normative dimension of this educational gap is significant. Medical curricula and continuing professional development programs reflect implicit priorities about which clinical areas merit emphasis. The relative neglect of infertility in rural health training suggests a normative assumption that fertility care is a specialised, urban concern rather than a core component of primary healthcare. This contrasts with other reproductive health issues, such as maternal mortality, HIV, and family planning, that receive substantial attention in rural health programming.

Participant 1 noted: “*Awareness to the staff members as well, because we get irritated sometimes*.” This comment reveals how provider attitudes, shaped by limited training and competing demands, can negatively affect patient experiences. When healthcare workers lack knowledge about infertility and its psychosocial impacts, they may dismiss or minimise patients’ concerns, contributing to stigma and discouraging careseeking.

International research confirms that workforce training gaps constitute a significant barrier to expanding ART access in underserved areas. In sub-Saharan Africa, the shortage of well-trained fertility specialists, particularly skilled embryologists, is identified as a critical limitation (2). In rural Australia, limited availability of trained providers and fragmented care pathways undermine access to sexual and reproductive health services (8). In China, the high costs associated with meeting stringent accreditation requirements, including advanced equipment and specialised personnel, create barriers to establishing ART services in underserved regions (15).

#### 3.2.3 Missed opportunities for preventive education

Participants emphasised that infertility education should not be confined to specialised fertility clinics but integrated into routine primary healthcare encounters. Participant 4 suggested:

“Yes, teach them... a person shouldn't come when they are a 40-yearold for the first time.”

This perspective positions infertility education as a continuum spanning prevention, early identification, and timely referral, rather than a discrete intervention limited to specialist care.

Routine clinical encounters, such as family planning consultations, STI screening, and general health visits, represent underutilised opportunities for fertility awareness education. By embedding fertility education within existing primary healthcare services, rural health systems could promote earlier presentation, reduce preventable causes of infertility, and improve timely referral without requiring substantial new resources.

This approach aligns with public health frameworks emphasising prevention and early intervention. Research demonstrates that many causes of infertility, including sexually transmitted infections, obesity, smoking, and delayed childbearing, are modifiable through education and behaviour change (19). Integrating fertility awareness into routine care could reduce future demand for expensive ART interventions while improving reproductive health outcomes.

However, implementing this approach requires *institutional change*. In terms of *regulation*, clinical protocols and performance metrics would need to incorporate fertility education as a standard component of primary care. *Normatively*, professional training and continuing education would need to equip providers with the knowledge and skills to deliver fertility counselling. *Cultural-cognitively*, assumptions about which health topics are appropriate for routine primary care discussions would need to expand to include fertility and reproductive life planning.

#### 3.2.4 Institutional analysis

The educational gaps documented in this theme reflect institutional priorities that have historically marginalised infertility within public health agendas, particularly in resource-constrained settings. Regarding regulation, the absence of national policies mandating infertility education or integrating fertility care into primary healthcare reflects a policy gap that perpetuates knowledge deficits (20). In contrast, other reproductive health issues, such as HIV prevention and maternal mortality reduction, benefit from well-funded, systematic education campaigns.

*Normatively*, the medical profession’s emphasis on acute, life-threatening conditions over chronic, quality-of-life concerns shapes training priorities and resource allocation. Infertility, while profoundly impactful for affected individuals, is not typically framed as a public health priority in the same way as infectious diseases or maternal mortality (12). This normative hierarchy influences what knowledge is transmitted in medical education and what services are prioritised in resource-constrained settings.

*Cultural-cognitively*, assumptions about fertility as a private, individual concern rather than a public health issue shape institutional responses. In many contexts, infertility is viewed as a personal misfortune rather than a healthcare need deserving of public investment (18). These cultural-cognitive frames influence policy decisions, resource allocation, and the development of education initiatives.

International evidence demonstrates that educational interventions can improve knowledge, reduce stigma, and promote earlier care-seeking for infertility. However, such interventions require institutional commitment and resources that are often lacking in rural and underserved settings (6, 8).

### 3.3 Theme 3: weak health system capacity undermining ART access

The third major theme concerns weak health system capacity as a significant barrier to ART access in rural South Africa. Participants identified multiple dimensions of system weakness, including workforce shortages, inadequate training, fragmented referral pathways, infrastructure limitations, and resource constraints that collectively undermine fertility care delivery. These challenges constrain the ability of rural healthcare facilities to provide effective infertility management and referral services.

#### 3.3.1 Workforce training and skills gaps

Participants emphasised the need for systematic training to equip rural medical officers with the knowledge and skills to manage infertility cases more effectively. Participant 6 stated:

“My first recommendation is to teach me how to do it. Second give me resources and improve referral pathways.”

This request reflects recognition that provider capacity is a critical bottleneck in service delivery.

Participant 11 advocated for decentralisation of skills:

“If more facilities are empowered through training, they can provide services without always referring patients.”

This perspective challenges the institutional logic that concentrates specialised knowledge in urban tertiary centres, suggesting instead that rural providers could be trained to deliver at least intermediate-level fertility interventions, reducing the burden on referral systems and improving access for rural patients.

The concept of task-shifting, training non-specialist providers to deliver services traditionally reserved for specialists, has been successfully applied in other areas of healthcare, particularly in resource-constrained settings (2). However, implementing task-shifting for fertility care requires institutional support, including training programs, clinical protocols adapted for rural settings, and mechanisms for ongoing supervision and quality assurance.

International evidence supports the potential of training interventions to expand access to ART. In China, recommendations include prioritising basic infertility diagnosis and cost-effective treatments at local facilities, utilising telemedicine for specialist consultation, and increasing the number of ART institutions in underserved provinces (15). In sub-Saharan Africa, expanding the availability of skilled embryologists and other fertility specialists is identified as essential for increasing ART service capacity (2). In rural Australia, recommendations include creating medical training opportunities for regional residents and providing incentives for doctors to relocate to rural health centres (5).

#### 3.3.2 Fragmented referral pathways and system navigation challenges

Clear, well-coordinated referral pathways were identified as essential for reducing delays and patient loss to follow-up. Participant 6 explained: “*So there is still a lot of stigma to get rid of A LOT. Except for physicians and patient education there must be improvement of referral pathways to facilities which have those or who offer those services*.” This comment links referral system improvement to broader efforts to reduce stigma and improve education, suggesting that these interventions must work in concert.

Fragmented referral pathways create multiple points of failure. Patients may not receive clear information about where to go, what documents to bring, or what to expect. Communication between referring and receiving facilities is often poor, leading to duplicate tests, delayed care, and frustrated patients. For rural patients already facing geographic and financial barriers, these system failures can be insurmountable. Research from diverse contexts documents how poor care coordination and fragmented pathways undermine access to reproductive health services. In the United States, Veterans seeking IVF report a lack of comprehensive care coordination and poor communication about benefits, particularly affecting those in rural areas (17). In Australia, rural women experience disrupted continuity of care due to rotational staffing models and limited service availability at regional clinics (5). In Brazil, the concentration of public ART services in the Southeast region and long waiting lists create access barriers for patients in other regions (20).

Improving referral pathways requires institutional interventions at multiple levels. In regulation, requirements mandate formal protocols and information systems to standardise referral processes and ensure communication between facilities. Normatively, professional standards should emphasise care coordination and patient navigation as core competencies. Organizationally, dedicated resources, such as patient navigators or care coordinators, may be needed to support patients through complex referral processes.

#### 3.3.3 Resource constraints and infrastructure limitations

Beyond training and referral systems, participants identified fundamental resource constraints that limit rural facilities’ capacity to provide fertility care. These include a lack of specialised equipment (such as ultrasound machines capable of detailed pelvic imaging), laboratory capacity for hormonal assays and semen analysis, and physical infrastructure suitable for fertility procedures.

Participant 12 noted:

“The male usually just does a sperm count only. There are challenges in doing the sperm counts because the closest centre will be in Empangeni or Richards Bay.”

This comment illustrates how even basic diagnostic tests may be unavailable locally, requiring patients to travel to access services that should be available at the primary care level.

Infrastructure limitations reflect regulative decisions about resource allocation and investment priorities. In many countries, public health systems are chronically underresourced, with rural facilities particularly disadvantaged (6, 20). The concentration of resources in urban tertiary centres reflects institutional logics that prioritise specialised, high-technology care over strengthening primary and secondary care.

International evidence demonstrates that infrastructure and resource constraints are major barriers to expanding ART access in underserved areas. In China, the high costs of meeting stringent accreditation requirements, including facility construction, advanced equipment, and specialised personnel, create barriers to establishing ART services in western provinces (15). In sub-Saharan Africa, lack of public funding for medical services and medication, combined with the largely privatised nature of ART provision, limits access for economically disadvantaged populations (2). In Brazil, insufficient public funding and scarcity of specialised human resources result in limited ART services outside major urban centres (20).

#### 3.3.4 Data systems and monitoring

While participants did not explicitly emphasise it, the absence of systematic data collection and monitoring systems represents a critical capacity gap. Without data on infertility prevalence, service utilization, referral outcomes, and treatment results, health systems cannot effectively plan services, allocate resources, or evaluate interventions. This data gap reflects broader weaknesses in health information systems in resource-constrained settings.

Strengthening data systems would enable evidence-based planning and policy development. For example, data on the geographic distribution of infertility cases, common etiologies, and referral patterns could inform decisions about where to invest in training, equipment, and service expansion. Data on patient outcomes after referral could identify gaps in care coordination and opportunities for improvement.

## 4 Discussion

This study provides empirical evidence of profound institutional barriers to ART access in rural South Africa, as experienced by medical officers working at the frontlines of service delivery. The findings reveal how regulative gaps, normative priorities, and cultural-cognitive assumptions embedded in healthcare systems create systematic disadvantages for rural populations seeking fertility care. Three key themes, limited scope of rural infertility services, inadequate patient and provider education, and weak health system capacity, illuminate the multidimensional nature of these barriers and point toward comprehensive institutional reforms needed to achieve reproductive justice.

### 4.1 Institutional barriers and reproductive inequity

The concentration of ART services in urban, private facilities documented in this study reflects institutional arrangements that systematically privilege affluent, urban populations while marginalising rural residents. This pattern is not unique to South Africa but represents a global phenomenon documented across diverse contexts (1, 13, 14, 22). From an Institutional Theory perspective, these geographic disparities reflect the interaction among the regulative, normative, and cultural-cognitive institutional pillars, which collectively shape healthcare access.

From an Institutional Theory perspective, the limited scope of rural infertility services reflects the interaction of all three institutional pillars.

Regulatory constraints on resource allocation and licensing requirements concentrate specialised services in urban tertiary centres. In China, for example, government policies limit the number of ART clinics per province and impose stringent accreditation requirements that favour large, well-resourced urban facilities (15). In Brazil, insufficient public funding and the concentration of services in the Southeast region leave rural and underserved areas with scarce or non-existent access (20).

Normatively, professional standards and clinical guidelines often assume access to specialised equipment and personnel that are unavailable in rural settings. The medical education system trains specialists for urban practice environments, with limited emphasis on adapting fertility care for resource-constrained contexts (2). This creates a normative gap where rural providers lack both the training and the institutional support to provide even intermediate-level fertility interventions.

Cultural-cognitively, taken-for-granted assumptions about rural healthcare capabilities shape what services are considered feasible or appropriate. The institutional logic that associates advanced reproductive technologies exclusively with urban, high-resource settings becomes self-fulfilling, as investment and innovation are directed toward urban centres. At the same time, rural facilities remain under-resourced and under skilled.

These institutional barriers are not unique to South Africa but reflect global patterns of healthcare inequality. Studies from diverse contexts, including China, the United States, Australia, Brazil, and other sub-Saharan African countries, document similar concentrations of ART services in urban areas and the resulting access disparities for rural populations (1, 2, 14, 22).

In terms of regulation, the absence of public funding for ART in South Africa, where only two medical aid schemes cover fertility treatments and services are concentrated in tertiary hospitals in major cities, creates financial and geographic barriers that are insurmountable for most rural residents. This contrasts with countries like Australia, where universal health insurance subsidises ART, yet geographic disparities persist due to clinic concentration in metropolitan areas (14). Even supportive regulatory frameworks cannot fully overcome geographic barriers when services remain centralised.

The South African context is particularly challenging given the dual healthcare system, in which a well-resourced private sector serves a minority of the population. In contrast, the public sector, serving the majority, is chronically under-resourced (3). The concentration of ART services in the private sector effectively excludes the majority of South Africans, particularly rural residents who depend on public healthcare. This institutional arrangement violates constitutional guarantees of equitable healthcare access and perpetuates reproductive injustice.

Normatively, the findings reveal how professional priorities and training systems contribute to disparities in access. Medical education in South Africa, as in many countries, emphasises specialist, hospital-based care over primary care and rural practice (2). Fertility medicine is taught as a subspecialty of obstetrics and gynaecology, with limited attention to how infertility can be addressed in resource-constrained primary care settings. This normative emphasis produces a workforce ill-equipped to provide even basic fertility care in rural contexts.

The relative neglect of infertility in public health programming reflects normative hierarchies that prioritise acute, life-threatening conditions over chronic, quality-of-life concerns (12). While maternal mortality, HIV, and infectious diseases receive substantial attention and resources in South African health policy, infertility remains marginalised despite its profound psychosocial impacts and the constitutional right to reproductive healthcare. This normative gap shapes resource allocation, training priorities, and service development in ways that disadvantage those seeking fertility care.

Cultural-cognitively, taken-for-granted assumptions about rural healthcare capabilities and patient demand shape institutional responses. The institutional logic that associates advanced reproductive technologies exclusively with urban, affluent populations becomes self-fulfilling, as investment and innovation are directed toward urban centres. At the same time, rural facilities remain under-resourced (5). Participant 4’s comment, suggesting that rural patients “*should not come when they are a 40-year-old for the first time,*” reflects internalised assumptions about appropriate timing and patient responsibility, potentially obscuring systemic barriers that prevent earlier access.

### 4.2 Education as institutional intervention

The educational gaps documented in this study, affecting both patients and providers, reflect institutional failures to prioritise fertility awareness and training. However, education alone is insufficient to address access barriers. As Institutional Theory emphasises, individual knowledge and attitudes are shaped by broader institutional contexts (9). Patients’ limited awareness of infertility causes and treatment options may partly reflect the limited integration of infertility education within public health programmes and reproductive health promotion initiatives. Previous studies have shown that low fertility literacy, stigma, and inadequate public awareness contribute to delayed care-seeking and reduced utilisation of fertility services, particularly in low- and middle-income countries (2, 25, 26).

Similarly, provider knowledge gaps reflect broader institutional priorities embedded within healthcare systems. From a regulative perspective, the absence of national policies mandating infertility education or integrating fertility care into primary healthcare contributes to persistent knowledge deficits among both patients and providers (20). Normatively, public health priorities and medical training programmes continue to emphasise maternal health, HIV/AIDS, and infectious diseases (12), while infertility receives comparatively limited attention despite its significant psychosocial and reproductive consequences. Consequently, many healthcare providers receive little formal preparation in infertility management, particularly within rural and primary healthcare settings. At the cultural-cognitive level, infertility is often viewed as a private or individual concern rather than a legitimate public health issue deserving of systematic investment and educational intervention (18). These institutional dynamics shape what knowledge is prioritised, transmitted, and acted upon within healthcare systems (6, 8), thereby perpetuating gaps in fertility awareness and access to care.

International evidence demonstrates that educational interventions can improve knowledge and reduce stigma, but their effectiveness depends on supportive institutional contexts (6, 8). In Brazil, despite growing awareness of ART, access remains severely limited for low-income populations due to insufficient public funding and service concentration in wealthy regions (20). This illustrates that education must be accompanied by structural reforms to expand service availability and affordability. The findings of this study are consistent with evidence from Australia, Brazil, China, and other low- and middle-income settings, which similarly report that infertility services are concentrated in urban centres and remain difficult to access for rural populations (14, 15, 20). However, while geographic disparities are evident across both high-income and developing contexts, the South African case is further complicated by the country’s dual healthcare system, in which advanced fertility services are concentrated in a largely private sector that remains inaccessible to many rural residents. This suggests that geographic exclusion in South Africa is reinforced not only by distance but also by economic barriers and uneven public-sector provision.

### 4.3 Health system strengthening and service delivery innovation

The health system capacity issues identified in this study, workforce shortages, infrastructure limitations, fragmented referral pathways, and resource constraints, require comprehensive institutional reforms. Participants’ calls for training, resources, and improved referral systems reflect recognition that individual provider efforts cannot overcome systemic barriers.

These capacity constraints reflect broader institutional weaknesses across the regulative, normative, and organisational dimensions of the healthcare system. From a regulative perspective, insufficient public investment in rural health infrastructure (15, 18), the absence of policies integrating fertility care into primary healthcare, and limited financial support for assisted reproductive technologies create structural barriers (11, 20) to service delivery. Normatively, medical training systems continue to prioritise specialist urban practice (5), contributing to workforce shortages and leaving rural providers with limited preparation for infertility management. Organisationally, the concentration of fertility services within specialised urban facilities, predominantly private, reinforces geographic and economic inequities (2), while fragmented referral systems further constrain access for rural populations. Collectively, these institutional arrangements limit the capacity of rural health systems to provide comprehensive fertility care and perpetuate disparities in access to reproductive health services.

Several service delivery innovations documented internationally offer potential models for improving ART access in rural South Africa:

- Telemedicine and remote consultation: Virtual consultations can reduce travel burdens and enable rural patients to receive specialist advice without travelling to urban centres (7, 15). However, telemedicine cannot fully replace in-person care for procedures that require physical presence, and infrastructure limitations (internet connectivity, equipment) may constrain its implementation in rural areas.
- Task-shifting and decentralisation: Training primary care providers to deliver basic fertility interventions, such as ovulation induction, hormonal treatments, and intrauterine insemination, could reduce dependence on urban specialists and improve access (2). This approach requires developing adapted clinical protocols, providing training and supervision, and ensuring quality assurance.
- Mobile clinics and outreach programs: Bringing services to rural communities through mobile units or periodic outreach visits could improve access to diagnostic services and consultations, though advanced procedures would still require referral to equipped facilities.
- Public-private partnerships: Collaborations between public health systems and private fertility clinics could expand service availability. However, careful attention to equity and affordability is essential to avoid perpetuating existing disparities.
- Financial assistance programs: Subsidies, insurance coverage, or public funding for ART could reduce economic barriers, though geographic barriers would remain unless accompanied by service decentralisation.

Implementing these innovations requires institutional commitment and resources. Regulatory measures mandating the integration of fertility care into primary healthcare, allocating public funding for ART services, and establishing quality standards for decentralised care are needed. Normatively, professional training systems must emphasise rural practice competencies and adapted service delivery models. Organizationally, health systems must invest in infrastructure, equipment, training, and care coordination mechanisms.

### 4.4 Reproductive justice and constitutional rights

The barriers documented in this study raise fundamental questions of reproductive justice and constitutional rights. Section 27 of the South African Constitution guarantees all citizens access to healthcare, including reproductive health services. However, the concentration of ART services in urban, private facilities effectively denies this right to rural residents and economically disadvantaged populations.

Reproductive justice frameworks emphasise that reproductive rights encompass not only the right to prevent pregnancy but also the right to have children and to parent in safe, supportive environments (12). Infertility represents a significant barrier to reproductive autonomy, and lack of access to fertility care constitutes a reproductive injustice, particularly when access is determined by geography and economic status rather than medical need.

International human rights frameworks recognise reproductive health as a fundamental right, yet implementation remains profoundly unequal (7, 12). The concentration of ART services in wealthy countries and urban areas within countries reflects broader patterns of global health inequality. Addressing these inequities requires political will, resource allocation, and institutional reforms that prioritise equity over commercial viability or administrative convenience.

### 4.5 Limitations and future research

This study has several limitations. First, the sample was limited to medical officers in one rural district, which may not represent experiences in other rural contexts or among other healthcare provider types (such as nurses or community health workers). Second, the study focused on provider perspectives; future research should examine patient experiences and perspectives to provide a more complete picture of access barriers. Third, the study did not quantify the magnitude of unmet need for fertility services in the study area, which would strengthen the case for policy intervention. Future research should examine: patient experiences and barriers to ART access in rural South Africa, cost-effectiveness of different service delivery models for expanding rural ART access, feasibility and acceptability of task-shifting fertility care to primary care providers, impact of telemedicine interventions on access and outcomes, policy analysis of regulatory and financing mechanisms to support equitable ART access, and comparative studies of successful models for expanding ART access in other resource-constrained settings.

### 4.6 Policy recommendations

Based on the findings, several policy recommendations emerge:

1. Develop a national policy framework for equitable ART access that recognises fertility care as essential healthcare and establishes targets for service availability in underserved areas.
2. Expand public funding for ART services through the National Health Insurance scheme or other mechanisms to reduce financial barriers for economically disadvantaged populations.
3. Integrate infertility education into primary healthcare by developing clinical protocols, training materials, and public health campaigns that promote fertility awareness and early intervention.
4. Invest in rural health infrastructure and workforce development by providing training for rural providers in basic fertility care, establishing telemedicine infrastructure for specialist consultation, and creating incentives for fertility specialists to practice in underserved areas.
5. Strengthen referral systems and care coordination by developing standardised referral protocols, implementing patient navigation programs, and establishing communication systems between rural and urban facilities.
6. Establish monitoring and evaluation systems to track infertility prevalence, service utilisation, referral outcomes, and treatment results to inform evidence-based planning and policy development.
7. Promote research and innovation in service delivery models adapted for resourceconstrained settings, including task-shifting, mobile clinics, and community-based interventions.

## 5 Conclusion

This study provides empirical evidence of profound institutional barriers to ART access in rural South Africa, revealing how regulative gaps, normative priorities, and culturalcognitive assumptions embedded in healthcare systems create systematic disadvantages for rural populations seeking fertility care. Medical officers in the UMhlabuyalingana district describe facilities capable of providing only preliminary diagnostic investigations, with no capacity for therapeutic interventions or advanced fertility treatments. This limitation reflects institutional arrangements that concentrate specialised reproductive healthcare in urban tertiary centres while relegating rural facilities to basic screening and referral functions.

Three key themes emerged from the analysis: limited scope of infertility management in rural facilities, inadequate patient and provider education, and weak health system capacity. These themes illuminate the multidimensional nature of access barriers and demonstrate that improving ART access requires comprehensive institutional reforms rather than merely individual-level interventions. The concentration of ART services in urban, private facilities effectively denies constitutional rights to healthcare for rural residents and economically disadvantaged populations, constituting a profound reproductive injustice.

Addressing these barriers requires political will, resource allocation, and institutional reforms across multiple levels. Regulatory interventions, including public funding for ART, policies mandating the integration of fertility care into primary healthcare, and resource allocation to rural health infrastructure, are essential. Normative changes, including medical education reform, development of adapted clinical protocols, and professional standards emphasising care coordination, are needed to shift priorities and practices. Cultural-cognitive shifts, challenging assumptions about rural healthcare capabilities and recognising fertility care as essential healthcare are necessary to transform institutional logics that perpetuate inequity.

The findings contribute to both theoretical understanding and practical policy development. From a theoretical perspective, the study demonstrates the utility of Institutional Theory for analysing healthcare access disparities, revealing how multiple institutional pillars interact to create and perpetuate inequity. From a policy perspective, the study provides evidence to support advocacy for reproductive justice and equitable healthcare access in South Africa and beyond.

Ultimately, achieving equitable ART access requires recognising fertility care as a fundamental component of reproductive healthcare, deserving of public investment and institutional support. Rural populations should not be left behind as medical technologies advance. Realising constitutional guarantees of healthcare access demands institutional transformation to ensure that geography and economic status do not determine reproductive opportunities and outcomes.

## Data Availability

The raw data supporting the conclusions of this article will be made available by the authors, without undue reservation.
